# Persistent Malnutrition and Associated Factors among Children under Five Years Attending Primary Health Care Facilities in Limpopo Province, South Africa

**DOI:** 10.3390/ijerph17207580

**Published:** 2020-10-19

**Authors:** Perpetua Modjadji, Josephine Mashishi

**Affiliations:** Department of Public Health, School of Health Care Sciences, Sefako Makgatho Health Sciences University, 1 Molotlegi Street, Ga-Rankuwa 0208, South Africa; matsiemashishi@gmail.com

**Keywords:** children, stunting, underweight, thinness, associated factors, primary healthcare facilities, South Africa

## Abstract

Despite years of interventions intended to reduce child malnutrition in South Africa, its negative effects, stunting in particular, persist mainly among children under five years old living in under-resourced regions. A cross-sectional study was conducted to determine the prevalence of malnutrition and associated factors among 404 children under age five attending childcare services with their mothers in selected healthcare facilities of Limpopo Province, South Africa. Anthropometry, socio-demographics and obstetric history were collected. Height-for-age, weight-for-age and body mass index-for-age Z-scores were used to determine stunting, underweight and thinness among children, respectively. Logistic regression analyses were performed to generate the factors associated with malnutrition. Stunting (45.3%) was the prevalent form of malnutrition among children under age five, affecting boys (51.7%) more than girls (38.8%) and children aged 12–23 months (62.4%) more than those <11 months old (40.1%), in addition to the overall prevalence of underweight (29.0%) and thinness (12.6%). Boys had increased odds of stunting (adjusted odds ratio, AOR = 2.07, 95% CI: 1.26–3.41, *p* = 0.004) and underweight (AOR = 2.17, 95% CI: 1.32–3.57, *p* = 0.002) than girls. Children aged 12–23 months were more likely to be stunted (AOR = 4.79, 95% CI: 2.36–9.75, *p* ≤ 0.0001) than children aged ≤11 months. Delayed introduction of solid foods increased the odds of stunting (AOR = 5.77, 95% CI: 2.63–12.64, *p* ≤ 0.0001) and underweight (AOR = 2.05, 95% CI: 1.08–3.89, *p* = 0.028). Children with normal birth weight were less likely to be thin (AOR = 0.42, 95% CI: 0.19–0.92, *p* = 0.029) and underweight (AOR = 0.34, 95% CI: 0.17–0.68, *p* = 0.003) than children who had low birth weight. Children whose mothers had obtained secondary school education (AOR = 0.39, 95% CI: 0.16–0.97, *p* = 0.044), and Grade 12 or post-Grade 12 education (AOR = 0.32, 95% CI: 0.12–0.83, *p* = 0.020) were less likely to be stunted than were children of mothers who had only primary school education. Suboptimal complementary feeding predisposed children to stunting and underweight. National nutrition programs should be context-specific to improve the introduction of complementary foods among children, especially in the remote and poor areas.

## 1. Introduction

Malnutrition has persisted over the past decades and continues to affect the lives of children, especially in the developing world [1,2,3]. In particular, undernutrition (i.e., stunting, underweight and wasting) indicates insufficient intake of energy and nutrients to meet an individual’s needs to maintain good health and is prevalent among children under the age of five years [4,5,6]. Of concern is stunting (i.e., linear growth retardation or low height-for-age) occurring primarily in the first two–three years of life and frequently related to repeated exposure to adverse economic conditions, poor sanitation and the interactive effects of poor energy and nutrient intakes and infection [7]. In addition, underweight (i.e., low weight-for-age) indicates a history of poor health or nutritional insult to the child, including recurrent illness and/or starvation, while thinness (i.e., low weight-for-height) is an indicator of wasting and is generally linked with recent illness and failure to gain weight or a loss of weight [7]. All forms of malnutrition predispose children to increased risk of infection, the likelihood to die from common diseases such as respiratory infections and diarrheal diseases, and delayed cognitive development, leading to low adult incomes, poor economic growth and intergenerational transmission of poverty [8,9].

In sub-Saharan Africa (SSA), the prevalence of stunting, underweight and wasting among children under age five has been estimated at 39%, 25% and 10%, respectively [4]. In South Africa, the Demographic and Health Survey (SADHS) has reported stunting (27%) as the most common manifestation of malnutrition affecting children under five years, with substantially low prevalence of underweight (5.9%) and wasting (2.5%). Major determinants of child malnutrition in SSA are poverty and socioeconomic inequality, which are rife in South Africa [10,11]. Children in the poorest wealth group had triple prevalence of stunting (36.3%) compared to those in the wealthiest group (12.5%) [12]. Additionally, factors associated with malnutrition among children under age five in African countries include the age of the child, gender, birth weight, birth order, maternal education, mother’s body mass index, clean water availability, hygiene and sanitation, family structure and family size [13,14,15].

The first 1000 days of life are critical for nutrition, and if malnutrition is not addressed by the age of five years, the child might not reach full potential in life [16]. To address child malnutrition in South Africa, the government introduced the Integrated Nutrition Programme in 1995, with the aim to guide health workers on health promotion and supplementary feeding to those who are malnourished or at risk of becoming malnourished and on rehabilitation of the malnourished individuals [17,18]. Further current intervention strategies in South Africa also allow for targeted supplementation in children aged 6–59 months for the purposes of preventing or treating moderate malnutrition as shown by the Roadmap for Nutrition 2013–2017 [19]. Despite these key interventions, stunted growth in particular has persisted for over two decades among children aged under five years in South Africa [20], especially in the poverty-stricken regions [21]. Limpopo Province is one of the nine provinces in South Africa and the poorest with the lowest per capita allocation for primary health care, despite having the highest child poverty rates [22]. Child malnutrition remains an ongoing crisis in the province, ranked third in the country in terms of hospital admission and deaths with severe malnutrition for children below five years [23].

During 2017/2018, approximately 11,000 children under five years were treated for severe acute malnutrition, which was an underlying cause of mortality in 30.9% of these children [24]. Research is limited on malnutrition among children under age five in the health facilities in Limpopo Province [25], compared to several community-based studies [26,27] and national studies, which included the province [28,29]. In view of this, the aim of the study was to determine the prevalence of malnutrition in the form of stunting, underweight and thinness and associated factors among children aged under five years in the healthcare facilities of Waterberg district, Limpopo Province of South Africa. The implications of this study may highlight a need to review the existing plan of action in addressing child malnutrition. Furthermore, there is a need to identify contextual strategies that consider the resource status of a setting as well as the cultural diversities in the country, bearing in mind that all forms of hunger and malnutrition must be ended by 2030 as one of the goals set by the United Nations [30].

## 2. Methods

### 2.1. Study Design

This was a cross-sectional study conducted in the Waterberg primary healthcare (PHC) facilities from July 2019 to December 2019. We used PHC facilities based on the evidence of malnutrition still observed among children under age five attending outpatient departments in facilities, as well as hospital admission and deaths resulting from severe acute malnutrition in Limpopo Province [23,25].

### 2.2. Study Setting

The study was conducted in Waterberg District, located in the western part of Limpopo Province, South Africa. The district has a population of 734,780 and is made up of six sub-districts, namely Bela-Bela, Lephalale, Modimolle, Mogalakwena, Mookgophong and Thabazimbi. The head count of children below five years of age seen in all PHC facilities of Waterberg District was estimated at 67,776 in the 4th quarter of 2015/16 [23]. PHC facilities within the six sub-districts are demarcated into ten local areas [31]. Waterberg district was selected as a study setting because data on the prevalence of stunting, underweight and thinness from PHC facilities are scarce.

### 2.3. Study Participants

The study included children who were below the age of five years, brought by their biological mothers to attend childcare services on routine immunization and nutritional status monitoring, offered in the ten selected PHC facilities of Waterberg District. These children were not sick at the time of study, based on their mothers’ reports, and were without physical disabilities that could compromise their stature.

### 2.4. Sample Size and Sampling Procedure

Rao software was used to calculate a sample size, taking into consideration the population size of 67,776 children, a 5% margin of error and 95% confidence level. A minimum representative sample of 378 was calculated and buffered with 10% to cater for non-response, and a sample size of 415 was obtained. One facility was randomly selected per local area, and ultimately ten PHC facilities were used in the study. On the day of data collection, after the researcher explained the purpose of the study to mothers in each selected facility, convenience sampling was used to select children whose mothers consented to participate. The difficulty in obtaining a random sample from outpatients waiting in queues for services in the facility informed the use of convenience sampling. Although convenience sampling is a non-probability sampling method, it is the most applicable and widely used method in clinical research [32]. In this study, children and their mothers were enrolled according to their availability and accessibility at the time of the study.

### 2.5. Data Collection

#### 2.5.1. Sociodemographic and Obstetric History

A structured interviewer-administered questionnaire was adapted from studies conducted in a population living in a similar socioeconomic context in Limpopo Province [21,33]. The questionnaire considered the UNICEF conceptual framework for malnutrition [34] and data were collected on socio-demography, obstetric history and children’s characteristics. The questionnaire was validated through content and face validity and a pilot study. Content validity was achieved by making use of experts in the field to ensure that the questionnaire items would adequately measure the constructs they were intended to assess, and whether the items were sufficient to measure the area of interest [35]. Face validity was ensured through experts’ judgement to make sure that the questionnaire would measure what it intended to measure. Independent translators who speak Sepedi as their mother tongue and are conversant with English did forward and backward translations of the questionnaire. An expert committee approved the final version of the translated questionnaire [35]. To make sure that the translated items retained the same meaning as the original items, and to ensure that there was no confusion regarding the translated questionnaire, a pilot study was conducted to pretest the questionnaire and determine its feasibility [35]. Prior to the pilot study, the research assistants who speak Sepedi were taken through the process of conducting preliminary interviews in a local language. During the pilot study, research assistants were assessed while administering a questionnaire to participants in a local language and taking anthropometric measurements. A pilot study was conducted among 38 mothers in one of the facilities that did not form part of the study, and the results were not included in the data analysis for the main study. Literature documents use of a small sample size of about 30 to 50 of the respondents in a pilot study [36]. After pretesting the questionnaire, there were no changes to the content except for minimal clarity of wording, and simplifying layout and style. The questionnaire was comprised of sociodemographic questions on the personal information of mothers such as age, marital status, education level, as well as household information regarding income, family size, head of household, availability of electricity and sanitation. In addition, obstetric history and children’s characteristics, including mother’s age at birth, obstetric complications, childbirth date, sex, birth order, birth weight and selected feeding practices, mainly on breastfeeding and the introduction of solid foods, were collected.

#### 2.5.2. Anthropometric Measurements

Anthropometric measurements of height and weight for each child were determined using WHO standard procedures [37]. Weights (W) of children were measured using an infant electronic digital weighing scale, while recumbent length (L) was measured using a non-stretch 150 cm tape measure. For malnutrition indicators, anthropometric measurements were converted to sex-specific Z-scores using WHO Anthro Software. The three indicators assessed were length/height-for-age (HAZ), weight-for-age (WAZ) and body mass index-for-age (BAZ) Z-scores for all the children. Children with HAZ, WAZ and BAZ between −2SD to +2SD were classified as normal; those with greater than +2SD were regarded as tall, overweight and obese; while Z-scores between −3SD to −2SD were classified as stunted, underweight and thin, respectively. Severe cases of stunting, underweight and thinness were classified as Z-scores below −3SD, while BAZ between +1SD and +2SD indicated a possible risk of overweight [37]. 

Weights for mothers were measured to the nearest 0.1 kg using a calibrated smart D-quip electronic scale, and heights to the nearest 0.1 cm using a stadiometer, according to WHO [38]. Three measurements were taken and the average weight (W) and height (H) were recorded. Body mass index (BMI) was calculated as the weight in kilograms divided by the height in meters squared (BMI (kg/m^2^) = weight (kg)/height (m^2^)). Normal BMI is within 18.5 to 24.99 kg/m^2^. Underweight is defined as BMI <18.5 kg/m^2^, overweight as BMI of 25 to 29.99 kg/m^2^ and obesity as BMI ≥30 kg/m^2^. Furthermore, the mothers’ waist (WC) and hip (HC) circumferences were measured to the nearest 0.1 cm using a non-stretchable plastic tape. Abdominal obesity was defined at WC ≥88 cm and waist–hip ratio (WHR) equal to or greater than 0.85 [39].

### 2.6. Statistical Analysis

Statistical analyses were performed using STATA 14 (StataCorp. 2015 *.Stata Statistical Software*: Release 14. College Station, TX, USA). Complete case analysis was used to identify participants with missing data during analysis. Skewness and kurtosis tests for normality were performed to check the distribution of data for children (i.e., age, weight, height, HAZ, WAZ and BAZ) and for mothers (i.e., age, weight, waist, hip and height). All variables had Chi (2) *p*-values less than 0.05, which indicated skewed data, except for the height of mothers. The medians for HAZ, WAZ and BAZ of the children were compared using the Mann–Whitney test by sex. The results are presented as median (interquartile range (IQR)). The Kruskal–Wallis test was used to compare the medians for HAZ, WAZ and BAZ by age, and the results are presented as median (interquartile range (IQR)). Following the Kruskal–Wallis test, the Dunn test was further applied for multiple pairwise comparison of medians, and Bonferroni adjusted *p*-values between groups are reported. A Chi-square test was used to compare the prevalence of malnutrition indicators, stratified by sex and age, and the results are presented as frequency (*n*) and percentage (%). Fisher’s exact was applied to variables with expected values less than five (5) in a cell. Univariate and multivariate logistic regression analyses were used to determine the association of malnutrition indicators with child and maternal variables. The purposeful selection process began with a univariate analysis of each variable. Any variable having a significant univariate test at some arbitrary level was selected as a candidate for the multivariate analysis. We based this on the Wald test from logistic regression and a *p*-value cut-off point of <0.20. A stepwise backward elimination procedure was used to eliminate confounders. Adjusted odds ratios (AOR) with a 95% confidence interval (CI) were generated and used to determine the independent strength of the relationship. Significance was considered at *p* < 0.05.

### 2.7. Ethics Statement

This study was conducted according to the guidelines laid down in the Declaration of Helsinki and all procedures involving human subjects were approved by Sefako Makgatho Health Sciences University Research and Ethics Committee (SMUREC) (SMUREC/H/45/2019: PG). Furthermore, this study received permission from the Department of Health in Limpopo Province, South Africa, and written consent was obtained from the mothers.

## 3. Results

### 3.1. Characteristics and Caring Practices of Children

In this study, 404 children aged under five years participated with their mothers, and the response rate was 97%. The median age (IQR) of the children was eight months (6.0; 13.8), ranging from 2 to 59 months. Although most of the children were still breastfed at the time of the study, mixed feeding was also practiced and some children were not yet on solid foods (Table 1).

### 3.2. Demography, Socioeconomy and Obstetric History of Mothers

The median age (IQR) of mothers was 28 years (23.0; 33.0) when presenting at the clinic. Mothers were characterized by singlehood (54.2%), unemployed (78.7%), lived on child grant (78%), but at least had secondary education (59.4%). Households with five family members and above were observed, and most had lower monthly income (Table 2). Some mothers reported to have had a history of obstetric complications, such as delivering premature and low birth weight newborns, iron deficiency anemia, gestational diabetes, pre-eclampsia and postmaturity C-section (not shown in the table).

### 3.3. Malnutrition Indicators among Children

Using the WHO classification of nutritional status [37], stunting (45.3%) was more prevalent among children, followed by underweight (29.0%) and thinness (12.6%). Boys were more affected by stunting (*p* = 0.002) and underweight (*p* = 0.004) compared to girls, while the prevalence of thinness was not significantly different (*p* = 0.776). It is noteworthy that some children had a growth problem and some were at a risk of overweight, while overweight and obesity were less prevalent (Table 3).

In Table 4, most children aged 12–23 months were stunted compared to <11 months old and ≥24 months (*p* = 0.031). The sample size for age groups of the children was not evenly distributed, especially in the group of children >24 months. This could be because of the schedule of the expanded immunization program occurring frequently from birth, in weekly intervals, until the children are 18 months. As a result, children aged ≥24 months visit the facilities less frequently until they reached five to six years of age [40].

### 3.4. Factors Associated with Stunting, Underweight and Thinness

In the bivariate logistic regression, stunting, underweight and thinness were associated with either of the following; child’s age and sex, mother’s BMI, WC, marital status, mother’s education level, household size, parity, birth order, birth weight, time of initiated breastfeeding after birth and introduction of solid foods (*p* < 0.20). After controlling for potential confounding, the final multivariate logistic regression analyses showed that stunting was associated with child’s age and sex, mother’s education level and introduction of solid foods. Underweight was associated with child’s sex, birth weight and introduction of solid foods, while thinness was associated with birth weight (Table 5).

## 4. Discussion

This paper determined the prevalence of malnutrition and associated factors among children under age five attending PHC facilities in Waterberg district of Limpopo Province, South Africa. Stunting was the commonest form of malnutrition among children, followed by underweight and then thinness. The reported prevalence of stunting in the current study is almost similar to the prevalence reported among children in the Central Region of Limpopo Province in South Africa over a decade ago [41], indicating persistent stunting. Together with underweight and thinness, stunting has been reported among children under five years of age in various parts of the country [26,29,42] and other developing countries [43,44,45].

Delayed introduction of solid foods was associated with stunting and underweight among children in this study. Persistent stunting and underweight among children under five years of age in the current study could be due to inappropriate food supplementation during the weaning period when infants should undergo a transition from exclusive breastfeeding to including complementary foods in their diet. Impaired linear child growth (i.e., stunting) and the risk of malnutrition are worsened by continued breastfeeding not accompanied by adequate complementary feeding at the appropriate age, due to nutritional demand [46,47,48]. Besides delayed introduction of solid foods, most mothers in the current study reported practicing mixed feeding. Mixed feeding is the most common infant feeding practice in developing countries [49,50], including South Africa, and has health impacts on the child’s health due to exposure to the risk of diarrhea and malnutrition [51,52].

South Africa is not on track to meet the global target for exclusive breastfeeding; however, the SADHS (2016) has reported some notable improvements in exclusive breastfeeding among infants less than six months of age [18]. Then again, complementary feeding practices in South Africa are suboptimal and the action plan for complementary feeding receives little attention and resourcing, hence appropriate action is needed to improve this situation [53]. The Roadmap for Nutrition 2013–2017 in South Africa has reported supplementation in children aged 6–59 months for the purposes of preventing or treating moderate malnutrition [19].

Literature documents that mothers may prolong the duration of breastfeeding, especially if they do not know when to commence giving complementary food or due to poverty-related barriers to adequate complementary feeding [54]. Some of the reported common practices by mothers during the complementary feeding period are based on the types of food given to infants and the adequacy and frequency of feeding. Additionally, food restrictions due to cultural practices, unhygienic bottle feeding practices, food handling or preparation and responsive breastfeeding are also issues of concern during the complementary feeding period for children [55].

The current population was characterized by poor socioeconomic status based on the prevalence of unemployment, dependence on child social grants and singlehood with no spousal support, which may lead to food unavailability in households. In the context of food insecurity when adequacy and accessibility of food are impaired, the mother’s decision-making ability for effective infant feeding is also disrupted [56]. Complementary feeding cannot be addressed in isolation from the promotion of exclusive breastfeeding during the first six months, which is critical for survival and provides the foundation for healthy growth in early infancy as well as continued breastfeeding to age two years or beyond [57]. The conceptual framework on childhood stunting indicates a need to strengthen the complementary feeding component of infant and young child feeding [57]. The WHO recommends that the introduction of complementary foods to infants should be timely, adequate, safe and appropriate to reduce the prevalence of stunting in the second year of a child’s life [58,59].

Several low- and-middle-income countries, including Ethiopia, Nepal and Senegal, have dramatically reduced child stunting [60]. Strong contributors to change were improvements in maternal education, maternal nutrition, maternal and newborn care, and reductions in fertility or reduced inter-pregnancy intervals [60]. Therefore, there is a need to educate mothers regarding care, breastfeeding and transition to complementary feeding, in addition to family planning and addressing the modifiable vulnerabilities of poor socioeconomic status in South Africa. Appropriate breastfeeding and complementary feeding practices are considered fundamental to children’s survival, growth and development [60]. Researchers have reported that appropriate complementary feeding practices are associated with better household wealth, higher maternal education, child’s sex and age, low parity, maternal occupation, urban residence, knowledge and frequency of complementary feeding, receiving feeding advice and immunizations, among other factors [61].

The current study further showed that stunting was associated with a child’s sex and age and maternal education, while underweight was associated with a child’s sex and birth weight, and thinness was associated with birth weight. The biological differences of stunting among boys and girls have been attributed to morbidity during early life [61]. The difference in length among children has been ascribed to increase with age starting from 12 months until the child reaches two years of age [62], and this could be a reason for the likelihood of stunting as children age in the current study. As well, there is a notion that mothers who are literate may have more information for implementing optimal improved infant and young child feeding [63,64]. In addition, children who were born with a normal birth weight were less likely to be underweight. Weight at birth is an intergenerational issue dependent on an interplay of various factors, including maternal undernutrition, intrauterine growth, gestation at birth, birth spacing and order and maternal age [65].

Persistent early life malnutrition has been implicated to fuel the burden of non-communicable disease, an emerging public health concern in South Africa [66]. Similar to earlier suggestions [60], a multi-sectoral approach to reduce child stunting is an urgent call in South Africa to end all forms of malnutrition by 2030 as this is essential for Sustainable Development Goal 2. Addressing the challenges of child undernutrition in South Africa should carefully consider inadequate dietary intake, household food insecurity, inadequate care and feeding practices, an unhealthy household environment and inadequate health services. In addition, the basic causes include widespread poverty, increasing inequality and suboptimal public services, rooted in an inequitable economic system, as outlined in the UNICEF framework [67].

## 5. Limitations and Strengths

We acknowledge the limitation of using a cross-sectional study design, which could only report on inferences and not on causality. The other limitation is that we used convenience sampling. While our sample may not be representative of children who attended the PHC in Waterberg District of Limpopo Province, the large sample size used in our study provides valuable and useful information for comparisons with other published studies from developing countries. Missing data were excluded using complete case analysis, a commonly used method, although its limitations of affecting sample size and leading to reduced statistical efficiency of estimates while increasing the potential for bias have been reported [68]. Certainly, the inefficiency in our study might be less, since multiple missing values occurred within the same questionnaires (*n* = 11) [69]. We acknowledge the limitation of not testing the reliability of the questionnaire statistically, as one of the characteristics of a valid questionnaire [70], which was not done in the original studies [21,33] from where we adapted the questionnaire used in the current study. The questionnaire was, however, piloted before use to ensure its reliability. Recall and reporting biases from mothers on obstetric information, children’s feeding indicators and their history of illness events happening in the past are also acknowledged. Nonetheless, the adapted questionnaire was previously used in the population with a similar socioeconomic context [21,33], and was validated through content and face validity, use of independent translators for forward and backward translations, as well as by an expert committee.

## 6. Conclusions

Persistent malnutrition, in particular stunting and underweight, among children under five years of age in Waterberg district of Limpopo Province, are a public health concern. In addition to other factors associated with malnutrition indicators, delayed introduction of solid foods during the complementary feeding period predisposed children to stunting and underweight more so than children who had optimal complementary feeding. These findings clearly indicate a need to improve the introduction of complementary foods among children, through national nutrition programs that are context-specific, especially in the remote and poor areas. Moreover, modifiable maternal vulnerabilities such as a poor socioeconomic status should be considered in the intervention programs.

## Figures and Tables

**Table 1:** Characteristics and caring practices of children under five years of age (*n* = 404).

Variables	Category	Frequency	Percentage
Child sex	Girls	203	50.2
	Boys	201	49.8
Child age (months)	≤11	272	67.3
	12–23	101	25.0
	≥24	31	7.7
Birth order	First	140	34.7
	Middle	5	1.2
	Last	259	64.1
Birth weight	<2.5 kg	51	12.6
	≥2.5 kg	353	87.4
Term of pregnancy	Premature	28	6.9
	Full-term	268	91.1
	Post-term	8	2.0
Breastfed	Yes	384	95.1
	No	20	4.9
Time breastfed after birth	Within 1 h	325	84.6
	Between 1–24 h	54	14.1
	After 24 h	5	1.3
Length breastfed	<6 months	41	10.7
	Between 6–12 months	40	10.4
	>12 months	50	13.0
	Still breastfed	253	65.9
Introduction of solid foods	<6 months	86	21.3
	Between 6–12 months	182	45.1
	Delayed ^#^	136	33.7
Mixed feeding	Yes	268	66.3
	No	136	33.7

^#^ delayed introduction of solid foods means introduction only after 12 months of age.

**Table 2:** Characteristics of mothers of children under five years of age.

Variables	Categories	Frequency	Percentage
Mothers’ age (years) when presenting at the clinic	<25	142	35.2
	25–34	198	49.0
	≥35	64	15.8
Mothers’ age (years) when participating child was born	<25	157	38.9
	25–34	187	46.3
	≥35	60	14.8
BMI (kg/m^2^)	Underweight	19	4.7
	Normal	128	31.7
	Overweight	113	28.0
	Obesity	144	35.6
WC (cm)	Normal	210	52.0
	Abdominal obesity	194	48.0
WHR	Normal	255	63.1
	Abdominal obesity	149	36.9
Marital status	Single	219	54.2
	Ever married	86	21.3
	Cohabiting	99	24.5
Employed	Yes	86	21.3
	No	318	78.7
Education level	Primary school	41	10.2
	Secondary school	240	59.4
	Grade 12 and post	123	30.4
Receiving child grant	Yes	315	78.0
	No	89	22.0
Household head	Self	44	10.9
	Spouse	134	33.2
	Parents/grandparents	186	46.0
	Relative	40	9.9
Household income	<$60.14	15	3.7
	$60.14–$300.68	241	59.7
	>$300.68	148	36.6
Family size	1–4	257	63.6
	≥5	147	36.4
Dwelling place	Brick	234	57.9
	Non-brick	170	42.1
Electricity	Yes	370	91.6
	No	34	8.4
Has refrigerator	Yes	77	19.1
	No	327	80.9
Access to water	Yes	313	77.5
	No	91	22.5
Type of toilet	Flush	172	42.6
	Pit	232	57.4
Parity	1–2	247	61.1
	≥3	157	38.9
Obstetric complications	Yes	151	37.4
	No	253	62.6

BMI, body mass index; WC, waist circumference; WHR, waist–hip ratio.

**Table 3:** Comparison of medians and prevalence of malnutrition indices among children by sex.

Malnutrition Indicators	All	Boys	Girls	*p*-Value
	*n* = 404	*n* = 203	*n* = 201	
HAZ, median (IQR)	−1.74 (−3.32; 0.73)	−1.55 (−2.93; 0.92)	−2.19 (−3.58; 0.68)	0.127
Normal, *n* (%)	143 (35.4)	57 (28.1)	86 (42.8)	
Stunting, *n* (%)	183 (45.3)	105 (51.7)	78 (38.8)	0.002 *
Tallness, *n* (%)	78 (19.3)	41 (20.2)	37 (18.4)	0.069
WAZ, median (IQR)	−0.91 (−2.34; 0.85)	−0.73 (−1.93; 0.89)	−1.13 (−2.93; 0.7)	0.061
Normal, *n* (%)	190 (47.0)	83 (40.9)	107 (53.2)	
Underweight, *n* (%)	117 (29.0)	71 (35.0)	46 (22.9)	0.004 *
Growth problem, *n* (%)	97 (24.0)	49 (24.1)	48 (23.9)	0.272
BAZ, median (IQR)	−0.31 (−1.31; 0.83)	−0.28 (−1.19; 0.9)	−0.43 (−1.35; 0.71)	0.527
Normal, *n* (%)	262 (64.9)	133 (65.5)	129 (64.2)	
Thinness, *n* (%)	51 (12.6)	27 (13.3)	24 (11.9)	0.776
Overweight risk, *n* (%)	43 (10.6)	17 (8.4)	26 (12.9)	0.172
Overweight, *n* (%)	33 (8.2)	15 (7.4)	18 (9.0)	0.565
Obesity, *n* (%)	15 (3.7)	11 (5.4)	4 (2.0)	0.113 ^a^

IQR, interquartile range; HAZ, height-for-age Z-score; WAZ, weight-for-age Z-score; BAZ, body mass index-for-age Z-score. Stunting is defined as HAZ < −2SD, underweight defined as WAZ < −2SD and thinness defined as BAZ < −2SD; * indicates significant difference and ^a^ indicates Fisher’s exact.

**Table 4:** Comparison of medians and prevalence of malnutrition indices among children by age.

Malnutrition Indicators	≤11 Months	12–23 Months	≥24 Months	*p*-Value
	*n* = 272	*n* = 101	*n* = 31	
HAZ, median (IQR)	−1.31 (−3.44; −3.77)	−2.52 (−3.31; −1.38)	−1.58 (−2.45; −0.79)	*p*_1_ ≤ 0.0001 *
				*p*_2_ ≤ 0.0001
				*p*_3_ = 0.573
				*p*_4_ = 0.023
Normal, *n* (%)	85 (31.3)	38 (37.6)	20 (64.5)	
Stunting, *n* (%)	109 (40.1)	63 (62.4)	11 (35.4)	0.031 *
Tallness, *n* (%)	78 (28.7)	0	0	≤ 0.0001 ^a,^*
WAZ, median (IQR)	−0.54 (−2.21; 1.86)	−1.43 (−2.47; −0.55)	−1.38 (−2.36; −0.07)	*p*_1_ = 0.001 *
				*p*_2_ = 0.001 *
				*p*_3_ = 0.275
				*p*_4_ = 1.000
Normal, *n* (%)	111 (40.8)	58 (57.4)	21 (67.7)	
Underweight, *n* (%)	75 (27.6)	34 (33.7)	8 (25.8)	0.407
Growth problem, *n* (%)	86 (31.6)	9(8.9)	2 (6.5)	≤0.0001 ^a,^*
BAZ, median (IQR)	−0.56 (−1.37; 0.6)	0.17 (−0.99; 1.18)	−0.33 (−1.59; 1.64)	*p*_1_ = 0.025 *
				*p*_2_ = 1.000
				*p*_3_ = 0.007 *
				*p*_4_ = 1.000
Normal, *n* (%)	183 (67.3)	62 (61.4)	17 (54.8)	
Thinness, *n* (%)	39 (14.3)	9 (8.9)	3 (9.7)	0.622 ^a^
Overweight risk, *n* (%)	24 (8.8)	13 (12.9)	6 (19.4)	0.112 ^a^
Overweight, *n* (%)	18 (6.6)	12 (11.9)	3 (9.7)	0.177 ^a^
Obesity, *n* (%)	8 (2.9)	5 (5.0)	2 (6.5)	0.231 ^a^

IQR, interquartile range; HAZ, height-for-age Z-score; WAZ, weight-for-age Z-score; BAZ, body mass index-for-age Z-score. Stunting is defined as HAZ < −2SD, underweight defined as WAZ < −2SD and thinness defined as BAZ < −2SD; * indicates significant difference, ^a^ indicates Fisher’s exact; *p*_1_ = among the three groups, *p*_2_ between ≤11 months and 12–23 months, *p*_3_ between ≤11 months and ≥24 months, and *p*_4_ between 12–23 months and ≥24 months.

**Table 5:** Association of malnutrition indicators with selected independent variables.

Variables	AOR	95% CI	*p*-Value
**Stunting**			
*Child’s sex*			
Girls	1 (Reference)		
Boys	2.07	1.26–3.41	0.004 *
*Child’s age*			
<11 months	1 (Reference)		
12–23 months	4.79	2.36–9.75	≤ 0.0001 *
≥24 months	1.50	0.57–3.94	0.414
*Mother’s education level*			
Primary school	1 (Reference)		
Secondary school	0.39	0.16–0.97	0.044 *
Grade 12/post-Grade 12	0.32	0.12–0.83	0.020 *
*Introduction of solid foods*			
<6 months	1 (Reference)		
6–12 months	0.63	0.32–1.27	0.197
Delayed ^#^	5.77	2.63–12.64	≤ 0.0001 *
**Underweight**			
*Child’s sex*			
Girls	1 (Reference)		
Boys	2.17	1.32–3.57	0.002 *
*Birth weight*			
<2.5 kg	1 (Reference)		
≥2.5 kg	0.34	0.17–0.68	0.003 *
*Introduction of solid foods*			
<6 months	1 (Reference)		
6–12 months	0.74	0.38–1.47	0.395
Delayed ^#^	2.05	1.08–3.89	0.028 *
**Thinness**			
*Birth weight*			
<2.5 kg	1 (Reference)		
≥2.5 kg	0.42	0.19–0.92	0.029 *

^#^ delayed introduction of solid foods means introduction only after 12 months of age in this study. * significant association (*p* < 0.05).
